# Delayed biliary hemorrhage following transjugular intrahepatic portosystemic shunt

**DOI:** 10.1055/a-2891-5998

**Published:** 2026-07-21

**Authors:** Haihang Nie, Fan Wang, Baotakuzi Ailijiang, Feng Zhou, Qiu Zhao, Hongling Wang, Liping Chen

**Affiliations:** 1 Department of Gastroenterology 74547 Tianyou Hospital, Affiliated to Wuhan University of Science and TechnologyWuhanChina; 2 Department of Gastroenterology 89674 Zhongnan Hospital of Wuhan UniversityWuhanChina; 3 89674 Hubei Provincial Clinical Research Center for Intestinal and Colorectal DiseasesWuhanHubeiChina; 4 Hubei Key Laboratory of Intestinal and Colorectal Diseases 89674 Zhongnan Hospital of Wuhan UniversityWuhanHubeiChina

A 44-year-old man was admitted with a 9-day history of hematochezia and hematemesis occurring 3 hours prior to admission. He had a 15-year history of hepatitis B–related liver cirrhosis and had undergone splenectomy 13 years earlier. On admission, laboratory testing revealed a hemoglobin level of 74 g/L. Abdominal computed tomography (CT) demonstrated esophageal varices and dilation of the portal vein. After 2 days of medical therapy, a transjugular intrahepatic portosystemic shunt (TIPS) was performed to relieve portal hypertension ( Fig. 1 ). On postoperative day 7, the patient developed abdominal pain and recurrent hematochezia. Gastroscopy revealed fresh blood clots at the duodenal papilla, raising suspicion of post-TIPS hemobilia. Endovascular intervention was subsequently performed, with coil embolization of the gastroduodenal artery ( Fig. 2 ).

**Fig. 1:**
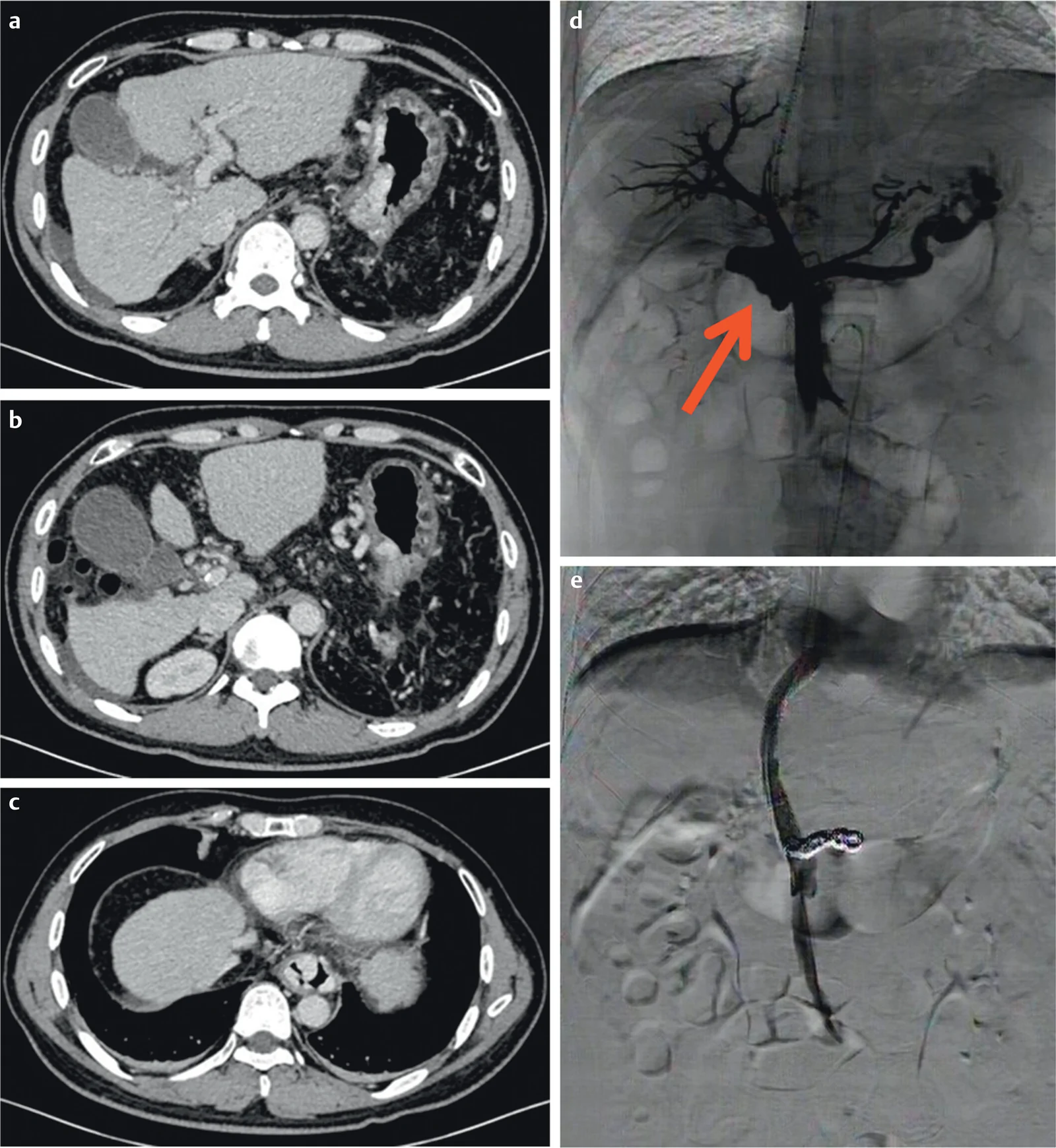
A transjugular intrahepatic portosystemic shunt (TIPS) procedure. ( **a** – **c** ) Abdominal computed tomography showing liver cirrhosis, esophagogastric varices, and portal vein dilatation with a small mural thrombus. ( **d** and **e** ) Puncture of the left branch of the portal vein via the right hepatic vein to establish the shunt tract.

**Fig. 2:**
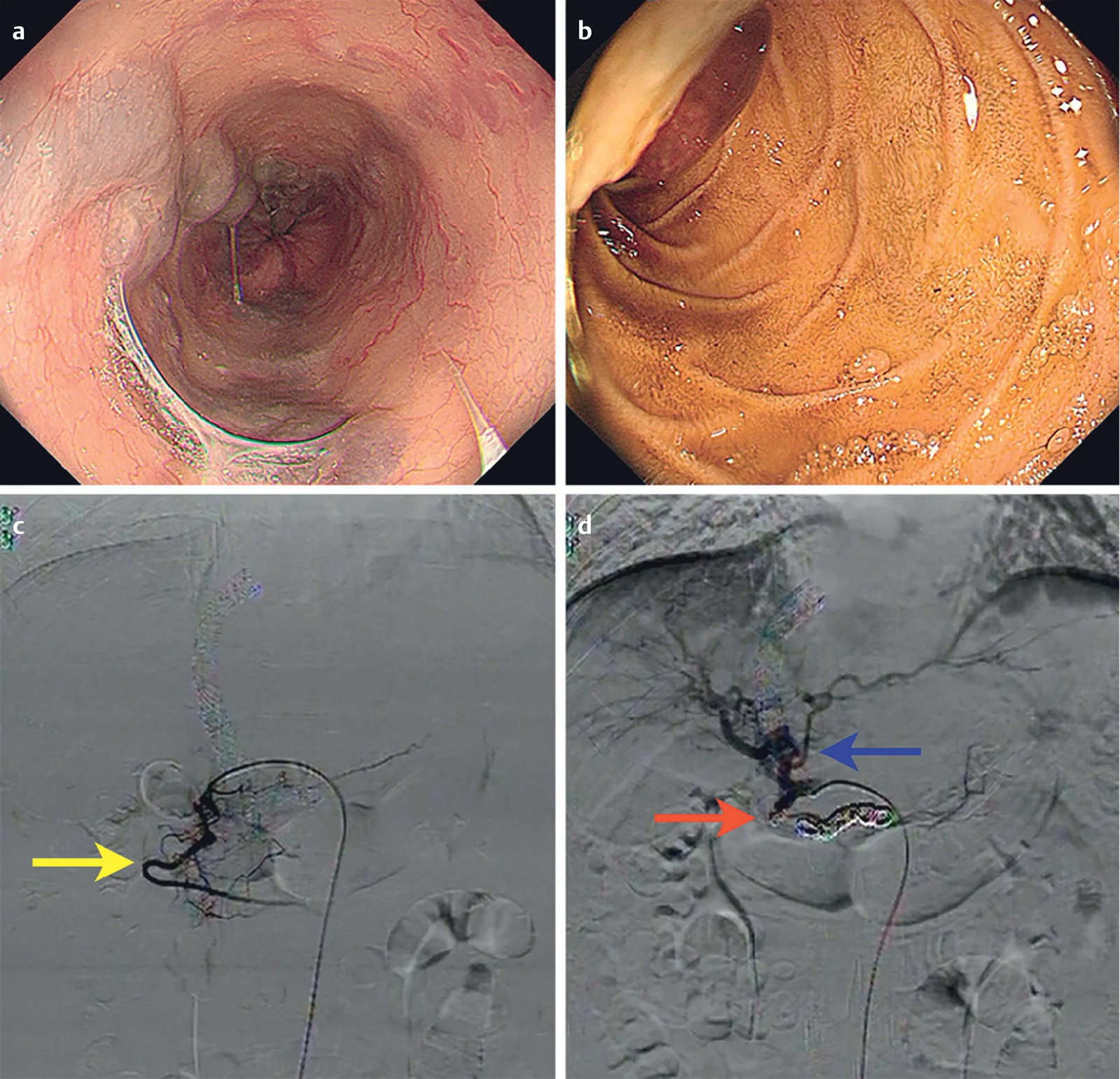
Angiographic embolization of the gastroduodenal artery. ( **a** ) Esophageal varices. ( **b** ) Fresh blood clots observed at the duodenal papilla. ( **c** and **d** ) Coil embolization of the gastroduodenal artery. Yellow arrows indicate the gastroduodenal artery; blue arrows indicate the proper hepatic artery; and red arrows indicate the embolization site.

Five days later, the patient’s symptoms worsened. Repeat abdominal CT showed gallbladder enlargement with intraluminal hemorrhage. Percutaneous transhepatic gallbladder drainage (PTGD) was performed, yielding bloody bile, and the patient’s symptoms were temporarily relieved ( Fig. 3 ). Five days after PTGD, the hemoglobin level had decreased to 48 g/L. To localize the bleeding source, endoscopic retrograde cholangiopancreatography with cholangioscopy (eyeMAX, 9-Fr; Micro-Tech (Nanjing) Co., Ltd, Nanjing, China) was performed for the direct visualization of the intrahepatic bile ducts. 1​ No bleeding source was identified in the right hepatic duct; however, fresh blood clots and a definite bleeding point were observed at the entrance of the left hepatic duct. A covered metal stent was placed in the left hepatic duct to achieve compression hemostasis, and a plastic stent was inserted into the right hepatic duct for biliary drainage ( Fig. 4 ; Video 1 ). During a 6-month follow-up period, no further episodes of abdominal pain or hematochezia were observed. This case highlights an effective endoscopic management strategy for secondary hemobilia following gastrointestinal interventional procedures.

**Fig. 3:**
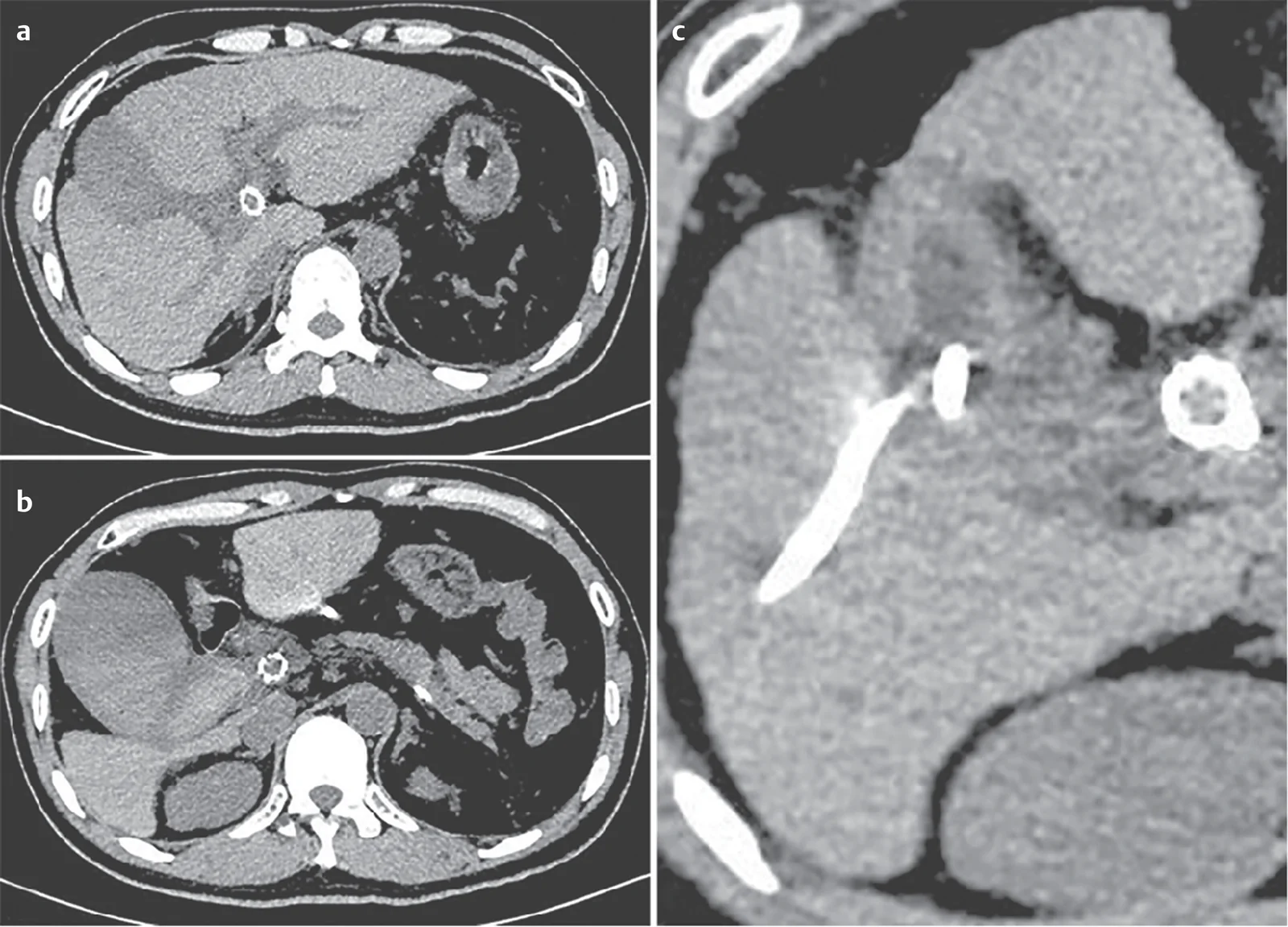
PTGD. ( **a** and **b** ) Markedly enlarged gallbladder with intraluminal hemorrhage. ( **c** ) Percutaneous gallbladder puncture and drainage revealing hemorrhagic bile. PTGD, percutaneous transhepatic gallbladder drainage.

**Fig. 4:**
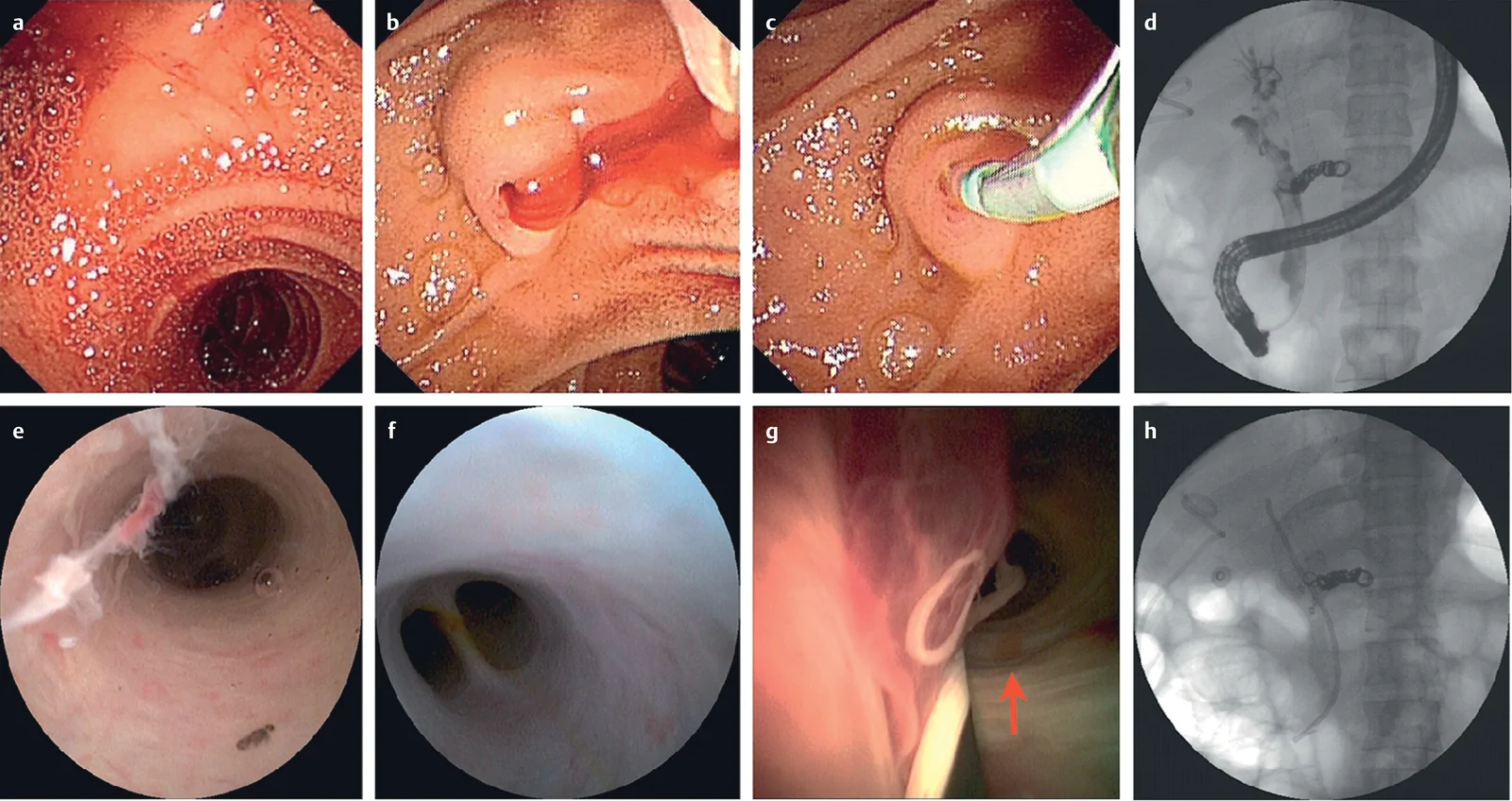
Cholangioscopy-guided management of intrahepatic biliary bleeding. Cholangioscopic examination of the intra- and extrahepatic bile ducts was performed. A narrowed orifice of the left hepatic duct was identified; under guidewire guidance, the cholangioscope was advanced, revealing an active bleeding site. A metal stent was subsequently placed to achieve hemostasis by compression.

**Video 1** Cholangioscopy-guided management of intrahepatic bile duct bleeding following transjugular intrahepatic portosystemic shunt.


Endoscopy_UCTN_Code_CCL_1AZ_2AO

## Notice

This article was changed according to the erratum on August 03, 2026.

## Erratum

In the above-mentioned article the author affiliations were presented incorrectly. Correct is: Nie Haihang 1; Wang Fan 2,3,4; Ailijiang Baotakuzi 2,3,4; Zhou Feng 2,3,4; Zhao Qiu 2,3,4; Wang Hongling 2,3,4; Chen Liping 2,3,4. 1 Department of Gastroenterology, Tianyou Hospital, Affiliated to Wuhan University of Science and Technology, Wuhan, China. 2 Department of Gastroenterology, Zhongnan Hospital of Wuhan University, Wuhan, China. 3 Hubei Provincial Clinical Research Center for Intestinal and Colorectal Diseases, Wuhan, Hubei, China. 4 Hubei Key Laboratory of Intestinal and Colorectal Diseases, Zhongnan Hospital of Wuhan University, Wuhan, Hubei, China. This was corrected in the online version on August 03, 2026.

## References

[1] Wang F Zhu Y Zhao Q Multifocal stenosis in purulent appendicitis with fecalith Endoscopy 2024 56 E108 E109 38307112 10.1055/a-2239-3401 PMC10837025

