# Correction: Delayed biliary hemorrhage following transjugular intrahepatic portosystemic shunt

**DOI:** 10.1055/a-2923-7492

**Published:** 2026-08-17

**Authors:** Haihang Nie, Fan Wang, Baotakuzi Ailijiang, Feng Zhou, Qiu Zhao, Hongling Wang, Liping Chen

**Affiliations:** 1 Department of Gastroenterology 74547 Tianyou Hospital, Affiliated to Wuhan University of Science and TechnologyWuhanChina; 2 Department of Gastroenterology 89674 Zhongnan Hospital of Wuhan UniversityWuhanChina; 3 89674 Hubei Provincial Clinical Research Center for Intestinal and Colorectal DiseasesWuhanHubeiChina; 4 Hubei Key Laboratory of Intestinal and Colorectal Diseases 89674 Zhongnan Hospital of Wuhan UniversityWuhanHubeiChina



In the above-mentioned article the author affiliations were not correctly presented. Correct is: Nie Haihang 1; Wang Fan 2,3,4; Ailijiang Baotakuzi 2,3,4; Zhou Feng 2,3,4; Zhao Qiu 2,3,4; Wang Hongling 2,3,4; Chen Liping 2,3,4 1 Department of Gastroenterology, Tianyou Hospital, Affiliated to Wuhan University of Science and Technology, Wuhan, China. 2 Department of Gastroenterology, Zhongnan Hospital of Wuhan University, Wuhan, China. 3 Hubei Provincial Clinical Research Center for Intestinal and Colorectal Diseases, Wuhan, Hubei, China. 4 Hubei Key Laboratory of Intestinal and Colorectal Diseases, Zhongnan Hospital of Wuhan University, Wuhan, Hubei, China. This was corrected in the online version on August 03, 2026.

